# Monitoring Internal Training Intensity Correlated with Neuromuscular and Well-Being Status in Croatian Professional Soccer Players during Five Weeks of the Pre-Season Training Phase

**DOI:** 10.3390/sports10110172

**Published:** 2022-11-02

**Authors:** Josip Maleš, Ibrahim Ouergui, Danijela Kuna, Frane Žuvela, Andrea De Giorgio, Goran Kuvačić

**Affiliations:** 1 Faculty of Kinesiology, University of Split, 21000 Split, Croatia; 2 High Institute of Sport and Physical Education of Kef, University of Jendouba, El Kef 7100, Tunisia; 3 Research Unit: Sport Sciences, Health and Movement, UR22JS01, El Kef 7100, Tunisia; 4 Faculty of Kinesiology, University of Osijek, 31000 Osijek, Croatia; 5 Faculty of Psychology, eCampus University, 22060 Novedrate, Italy

**Keywords:** training intensity, well-being, professional soccer

## Abstract

This study aimed to investigate the changes in internal training intensity, well-being, and countermovement jump (CMJ) performance and to determine their relationship across five weeks of the pre-season training phase in professional soccer players. A total of 22 professional male soccer players (age = 21.7 ± 4 years, body height = 185.9 ± 6.3 cm, body weight = 79 ± 6.3 kg, BMI = 22.8 ± 1.4 kg·m^−2^; VO_2max_ = 52.9 ± 3.2) from the Croatian Second League voluntary participated in this study. The players spent 2230 ± 117 min in 32 technical/tactical and strength/conditioning training sessions, mostly at the low intensity zone (61%), and played 8 friendly matches at a high intensity (>90%). A one-way repeated measure of analysis ANOVA revealed a significant difference between weeks in CMJ performance (F_(1,22)_ = 11.8, *p* < 0.001), with CMJ height in weeks 4 and 5 being likely to very likely higher than that noted in week 1. Moreover, significant differences between weeks were found in all internal training intensity measures (average [F_(1,22)_ = 74.8, *p* < 0.001] and accumulated weekly internal training intensity [F_(1,22)_ = 55.4, *p* < 0.001], training monotony [F_(1,22)_ = 23.9, *p* < 0.001], and training strain [F_(1,22)_ = 34.5, *p* < 0.001]). Likewise, differences were observed for wellness status categories (fatigue [F_(1,22)_ = 4.3, *p* = 0.003], sleep [F_(1,22)_ = 7.1, *p* < 0.001], DOMS [F_(1,22)_ = 5.7, *p* < 0.001], stress [F_(1,22)_ = 15.6, *p* < 0.001]), mood [F_(1,22)_ = 12.7, *p* < 0.001], and overall well-being status score (F_(1,22)_ = 13.2, *p* < 0.001). Correlation analysis showed large negative correlations between average weekly internal training intensity and fatigue (r = −0.63, *p* = 0.002), DOMS (r = −0.61, *p* = 0.003), and WBI (r = −0.53, *p* = 0.011). Additionally, fatigue was significantly associated (large negative correlation) with accumulated weekly internal training intensity (r = −0.51, *p* = 0.014) and training strain (r = −0.61, *p* = 0.003). Small, but non-significant, correlations were found between CMJ performance and wellness status measures. These findings highlight the utility and simplicity of monitoring tools to improve athletes’ performance.

## 1. Introduction

It is commonly known that the prescription for a successful training program requires the application of different training principles, including individualization, training intensity progression, and recovery to bring an equilibrium between training and rest periods to induce positive adaptations [1]. This fact requires that coaches not only concentrate on varying intensities and exercise modalities, but also that they constantly monitor the internal training intensities (ITI) to better understand this state of adaptation to avoid possible chronic fatigue [2]. While using several ITI monitoring tools to record data such as heart rate indices, blood lactate, oxygen uptake, and hormone levels is expensive and not available for all players and teams, the subjective measures of ITI-like scales and questionnaires are of great importance [3]. These scales and questionnaires have shown their effectiveness as quick and easy methods for detecting early signs of tiredness and monitoring well-being to optimize training and achieve high-level performance [4]. This may include the use of the rating of perceived exertion (RPE), developed by Borg [5] and adopted by Foster et al. [6] as s-RPE well-being indices including fatigue, stress, delayed onset muscle soreness, and sleep, which have shown significant interactions among these items [7,8] and that they were sensitive to increased training intensities, being factors that influence the technical-tactical performances of soccer players [4]. Moreover, in addition to these tools and to the aim to identify fatigue and adequate recovery during different training phases, several previous studies have examined the countermovement jump performance (CMJ) as a measure of neuromuscular fatigue [2,9,10].

Additionally, training intensities may vary according to the objectives of different phases of an entire annual training season, including the pre-season, during which coaches are striving to recondition players to attain the fitness levels of the past season, while during the in-season, maintaining the fitness levels achieved during the pre-season is the main objective [11]. This could apply to soccer players’ specific intensities in accordance with each distinct phase [12], which could therefore have a different impact on the players’ abilities to adapt, recover, and maintain their well-being. Specifically, previous researchers have assessed these variables in the pre-season [13,14,15] and the in-season [16,17,18], comparing both training periods [11,12,15]. Regarding the pre-season phase, it has been previously reported by Buchheit et al. [13] that during the 2 weeks of pre-season camp, there were significant day-to-day variations in training intensity (coefficient of variation [CV]= 66%) and wellness measures (6–18%), while the overall well-being did not change substantially throughout the camp, and that all well-being measures were related to delta training intensity. Furthermore, Selmi et al. [19] showed that 4 weeks of intense training during an early preparation period increased the training intensity and altered the technical performance, and that the well-being state was related to successful passes, interceptions, and ball losses during small-sided games. 

Due to the specificity of the pre-season training phase and its role in preparing players to handle the demands of the matches, and due to its specificity in terms of higher intensities compared to those in the in-season period [11,12,15], tracking players adaptations to this training period is of great interest, as it determines the success of the entire competitive season. Moreover, variations in well-being and training intensity outcomes, as well as relationships between them, must be determined when designing a training program for different season phases. Special consideration should be given to the effects of previously accumulated training intensity or neuromuscular fatigue on the fluctuations of the well-being status. Furthermore, investigations conducted under real-world conditions, and not simulations, are beneficial to coaches and team personnel because they provide a direct assessment of the effects of various training intensities. 

Considering all the aforementioned facts, the present study aimed to (a) investigate the changes of ITI, well-being status, and CMJ performance across 5 weeks of the pre-season training phase in professional soccer players and (b) examine the dose–response relationship between ITI and CMJ performance with the variations in well-being status measures.

## 2. Materials and Methods

### 2.1. Study Design

This was a 5-week longitudinal study that aimed to investigate the ITI, well-being, and CMJ responses of Croatian professional soccer players during the pre-season phase. In a consensus statement released by intensity monitoring experts, these variables were rated as practically useful and having medium to high reliability and validity [20]. After players returned from an off-season break, the study began at the beginning of July and ended in the first week of August. The players spent 5 weeks of the study in a controlled environment (training center). The team’s technical staff planned the entire training program during the pre-season phase. Training characteristics during this period are presented in Table 1. During the investigation, a total of 1764 observations were collected from training sessions and matches.

### 2.2. Participants

A total of 22 male professional soccer players (age = 21.7 ± 4 years, body height = 185.9 ± 6.3 cm, body weight = 79 ± 6.3 kg, BMI = 22.8 ± 1.4 kg·m^2^; VO_2max_ = 52.9 ± 3.2 mL kg^−1^ min^−1^) from the same team competing in the Croatian Second League participated in this study. Goalkeepers were excluded from the study due to their specific role on the team and their differing physical demands. Inclusion criteria to participate in the study were: (i) participation in at least 85% of the training sessions, and (ii) being healthy (no pain or injury) at the beginning of the pre-season phase. Participants provided their signed consent after being fully informed of the experimental procedures. 

### 2.3. Procedures

The team staff specified the training intensity, and the investigators had no influence on this. All training sessions began with a 30 min general warm-up composed of 5–10 min of jogging, 10–15 min of stretching, and 10–15 min of core exercises. The technical-tactical (TE-TA) training included game-based training drills and technical exercises that lasted 60 to 90 min per session. All training sessions were conducted in a controlled environment on the same regulation court. During the sessions, regular verbal support from the head coach and staff was provided. The training intensity was manipulated by changes in rules (e.g., number of players engaging in small-side games, court size etc.) and the work-rest ratio within and between exercises. Strength and conditioning (S&C) training sessions consisted of strength exercises with light to moderate intensity, plyometric drills, and specific repeated sprint bouts. The S&C sessions mainly targeted lower-body segments with some upper-body (e.g., bench press) exercises. The primary objective was to increase players’ strength and capacity for speed, agility, and endurance, as well as to enhance recovery between training sessions. Additionally, over the 5-week period, players participated in eight friendly matches. On each training day, daily subjective assessments of session rating of perceived exertion (sRPE) and well-being questionnaire were completed to assess ITI and well-being status, respectively. The sRPE assessment was previously introduced to all players during regular training sessions (in-season phase). The CMJ height was determined once a week prior to the first training session of the week. Additionally, the 30-15 Intermittent Fitness Test (30-15 IFT) was used to assess the ability to recover and repeat intermittent activity to calculate players’ maximum oxygen uptake (VO_2max_).

#### 2.3.1. Internal Training Intensity

The sRPE was quantified by using the Borg CR10 scale (1 = very, very easy and 10 = extremely hard) [21]. This method has been widely used in soccer for the calculation of training intensity [22,23]. Approximately 30 min following the completion of every training session and match, players were given a full view of the scale and were required to rate their perceived exertion by asking them: “How hard was your training session?” The questionnaire was administered by the same researcher throughout the whole period of investigation. To reduce the impact of hearing the ratings provided by other players, the scores were given individually to each player. To calculate ITI, the sRPE value reported by players was multiplied by the training duration (minutes). On days with two training sessions, the ITIs were summed. For further analyses, the following variables were calculated for each week: (a) meanITI—average ITI during each week, (b) sumITI—the accumulated ITI calculated as the summation of the training intensities of all training sessions during each week, (c) TM—training monotony calculated from the relation of meanITI to SD and the corresponding week, and (d) TS—training strain calculated by the multiplication of meanITI and TM. Moreover, the training sessions were divided into three intensity zones according to RPE data: low-intensity zone (Z1) ≤ 4 A.U.; 4 > moderate intensity zone (Z2) < 7 A.U.; and ≥7 high-intensity zone (Z3) [24].

#### 2.3.2. Well-Being Status 

To assess the daily well-being status of the players, a psychological questionnaire (WB) [25] consisting of five cognitive perceptions of fatigue, sleep, delayed onset of muscle soreness (DOMS), stress, and mood was applied. Players were required to rate each domain using a five-point Likert Scale (1—worst quality to 5—best quality) by asking them: “Please rate your level of fatigue, sleep quality, muscle soreness, mental stress, and mood.” The questionnaire was administered by the same researcher, and the responses were recorded individually to reduce the influence of other players on the scores. The well-being status index (WBI) represents the sum of all five domains (scores ranging from 5 to 25).

#### 2.3.3. Countermovement Jump

The CMJ was used to determine vertical jump height using a reliable and validated photoelectric cell system Optojump (Microgate srl, Bolzano, Italy). The players were instructed to conduct a downward movement followed by a full extension of the legs. With their hands fixed to their hips, players were verbally encouraged to jump as high as possible during each trial. Each player performed three jumps in a row, with a 15 s interval between the jumps. The highest jump of three attempts was used for further analysis. Two familiarization sessions were held seven days prior to the first week of the pre-season phase. The session started with a standard warm-up, after which participants practiced the jumps until they demonstrated consistent technique. The same experienced researcher performed all measurements.

#### 2.3.4. Aerobic Power Test

The 30-15_IFT_ was used to calculate the VO_2max_ of the players. On a 40 m straight runway, the test includes 30 s shuttle run alternated with 15 s passive rest periods. The running speed increases by 0.5 km/h every 45 s after the initial 8 km/h stage. An audible signal beeped at appropriate intervals to control the speed of the test, with players required to be within a 3 m tolerance zone at either end or the middle of the 40 m shuttle. Players were directed to go forward to the closest line at each extremity and the center of the shuttle at 20 m after successfully completing a level. Participants stopped voluntarily if they were completely worn out or if they were unable to complete the 2 m lines three times in a row. The speed of the last successfully completed stage was recorded as the maximum running speed (V_IFT_). The VO_2max_ was calculated by the following equation [26]:

VO_2max_ (ml kg^−1^ min^−1^) = 28.3 − (2.15 × 1) − (0.741 × age) − (0.0357 × weight) + (0.058 × age × V_IFT_) + (1.03 × V_IFT_)

The test was performed in the week preceding the first week of the pre-season phase.

#### 2.3.5. Anthropometric Measures

Body height was measured with Harpenden Portable Stadiometer 603 VR (Holtain LTD, Crosswell, UK). Body mass and BMI (body mass in kg/height in m^2^) were assessed by the Tanita diagnostic scale (BC 418). Standardized conditions were followed in terms of previous exercise, dietary intake, and skin temperature [27]. All measurements were taken by the same experienced researcher.

### 2.4. Statistical Analysis

Descriptive statistics were presented in the form of mean, standard deviation (SD), 90% confidence limits (90% CL), or the coefficient of variation (CV%). The normality and homogeneity of the data were analyzed using the Shapiro–Wilk test and the Levene test. After confirming normality and sphericity assumptions, a one-way repeated measures ANOVA was used to compare variables across the weeks. A Bonferroni post hoc test was conducted and Cohen’s d was calculated for pairwise comparisons. The magnitude of d was qualitatively interpreted using the following thresholds: <0.2, trivial; 0.2 to 0.6, small; 0.6 to 1.2, moderate; 1.2 to 2.0, large; and 2.0 to 4.0, very large [28]. Additionally, the magnitude-based inference was used to compare the differences in CMJ performance [29]. The chances of meaningful negative, trivial, or positive changes were determined qualitatively as follows: 1%, almost certainly not; 1–5%, very unlikely; 5–25%, unlikely; 25–75%, possible; 75–95%, likely; 95–99%, very likely; 99%, almost certainly. The true difference was deemed unclear if the chances of having poorer and better results were greater than 5%. To analyze the daily variations in ITI, WB scores, and weekly CMJ performance, terms such as “possibly” and “unclear” were used if the 90% confidence limits (CL) crossed one or both smallest worthwhile change boundaries (SWC; calculated by using 0.3 coefficient of variation), respectively. If the CL did not cross SWC boundaries, the effect was inferred a probable [30]. Pearson’s correlation coefficients were calculated to establish the correlations between internal ITI indices and well-being status measures. The threshold used to qualitatively assess the correlations was based on the following criteria [30]: 0.1, trivial; 0.1–0.3, small; 0.3–0.5, moderate; 0.5–0.7, large; 0.7–0.9, very large; 0.9, nearly perfect. The post hoc analysis to compute achieved power was conducted for a single within-subjects factor assessed over five time points and correlationsusing Power Analysis and Sample Size (PASS) software (version 15 LLC, Kaysville, UT, USA) and previously published recommendations [31]. The calculated power analyses (1-β) values were between 0.73 and 1 for all analyses with the sample size of twenty-two participants. The significance level was set at *p* < 0.05. All data were analyzed using SPSS 28.0 statistical software (SPSS, Chicago, IL, USA) and GraphPad Prism 9 (GraphPad Software, Inc., San Diego, CA, USA).

## 3. Results

**Figure 1:**
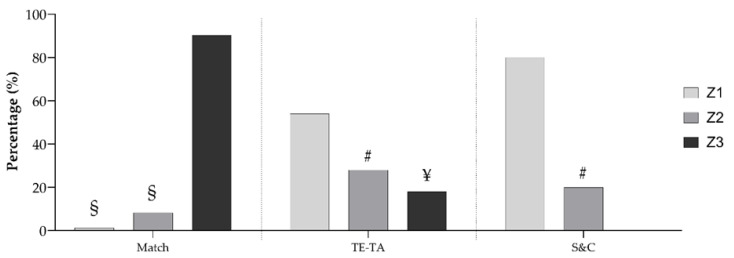
Percentage of the number of training sessions and matches carried out in the three intensity zones. Z1—low intensity zone; Z2—moderate intensity zone; Z3—high intensity zone; ¥—significantly lower than Z1 and Z2 at *p* < 0.001; #—significantly lower than Z1 at *p* < 0.001; §—significantly lower than Z3 at *p* < 0.001.

In total, players spent 2230 ± 117 min in 32 training sessions, of which 23 were TE-TA and 9 were S&C. The percentages of the number of training sessions and matches carried out in the three intensity zones are presented in Figure 1. The highest percentage of Z3 was determined during the matches, where Z1 and Z2 were lower than Z3. For TE-TA training session, the significant difference in training session distribution across intensity was also determined, in which Z2 and Z3 were lower than Z1. The players did not spend any of the S&C in Z3, but a significant number of sessions were performed in Z1. According to the distribution observed for the combined TE-TA and S&C training sessions, players performed approximately 61, 25, and 14% in low, moderate, and high intensity, respectively.

**Figure 2:**
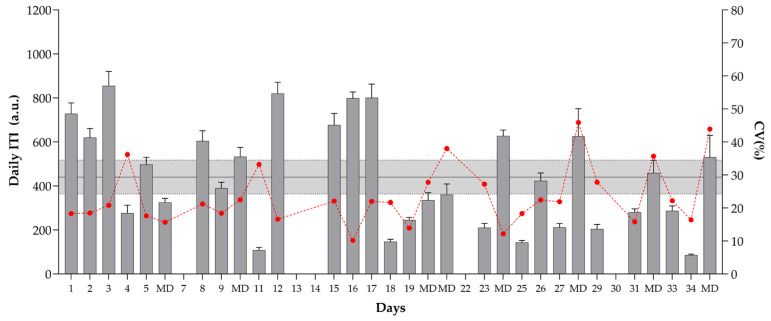
Daily internal training intensity (ITI) observed over the 35 days of the pre-season phase (5 weeks). The grey area represents the smallest worthwhile change (SWC = coefficient of variation × 0.3), and the error bars show 90% confidence limits (CL). If the CL crossed one or both SWC boundaries, the terms “possibly” and “unclear” were used, respectively. The red line represents within-days intensity variations (CV%). MD—match day.

**Figure 3:**
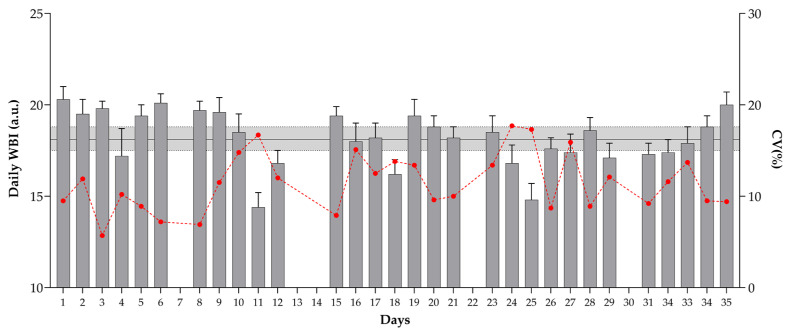
Daily well-being status index (WBI) observed over the 35 days of the pre-season (5 weeks). The grey area represents the smallest worthwhile change (SWC = coefficient of variation × 0.3), and the error bars show 90% confidence limits (CL). If the CL crossed one or both SWC boundaries, the terms “possibly” and “unclear” were used, respectively. The red line represents within-days well-being status variations (CV%).

The daily internal training intensity well-being status measures observed over the 35 days of the pre-season phase are presented in Figure 2 and Figure 3. The highest ITI was observed on day 3 and between days 12 to 17. Lower values were observed after training matches (2nd, 5th, and 6th match). Higher CVs were also noticed after the 6th, 7th, and 8th matches, with values ranging from 35.7 to 45.9%. The greatest positive change happened between days 11 and 12 (655.3%), while the greatest negative change appeared between days 17 and 18 (−81.7%). For the well-being status index, the highest values were observed in the first week of the investigation period (WBI: 18 to 20.3 a.u.). The highest CVs are found on days 24 and 25, while the daily changes are lower (values ranging from −22.1 to 19.6%) when compared with changes in ITI. Moreover, lower WBI values are noticeable after days of higher ITI (e.g., days 3, 17, 25).

**Figure 4:**
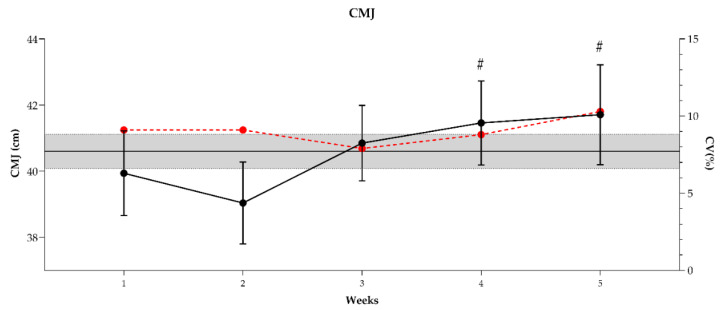
Countermovement jump (CMJ) height observed over the 5 weeks of the pre-season phase. The grey area represents the smallest worthwhile change (SWC = coefficient of variation × 0.3), and the error bars show 90% confidence limits (CL). If the CL crossed one or both SWC boundaries, the terms “possibly” and “unclear” were used, respectively. The red line represents within-weeks CMJ variations (CV%); #—significantly higher than W1.

Values of countermovement jump (CMJ) height over the five measurement points are presented in Figure 4. One-way repeated measures ANOVA revealed a significant difference between testing occasions (F_(1,22)_ = 11.8, *p* < 0.001). Additionally, compared with week 1 (baseline), CMJ height in weeks 4 and 5 were likely to be higher, respectively. Cohen d values ranged from 0.42 to 0.47, representing a small effect.

Table 2 represents ITI and well-being status measures across 5 weeks of investigation. Significant differences between weeks were found in all ITI measures (meanITI [F_(1,22)_ = 74.8, *p* < 0.001], sumITI [F_(1,22)_ = 55.4, *p* < 0.001], TM [F_(1,22)_ = 23.9, *p* < 0.001], and TS [F_(1,22)_ = 34.5, *p* < 0.001]). Post hoc analysis revealed that for meanITI, week 1 was statistically different from all other weeks. Almost similar was the result for sumITI, where week 1 was statistically different from all weeks, except week 3. For well-being status measures, one-way repeated measures ANOVA revealed a significant difference between weeks in all domains (fatigue [F_(1,22)_ = 4.3, *p* = 0.003], sleep [F_(1,22)_ = 7.1, *p* < 0.001], DOMS [F_(1,22)_ = 5.7, *p* < 0.001], stress [F_(1,22)_ = 15.6, *p* < 0.001]), and mood [F_(1,22)_ = 12.7, *p* < 0.001]), as well as overall WBI score (F_(1,22)_ = 13.2, *p* < 0.001). Post hoc analysis revealed that greatest differences between weeks were found for WBI.

**Figure 5:**
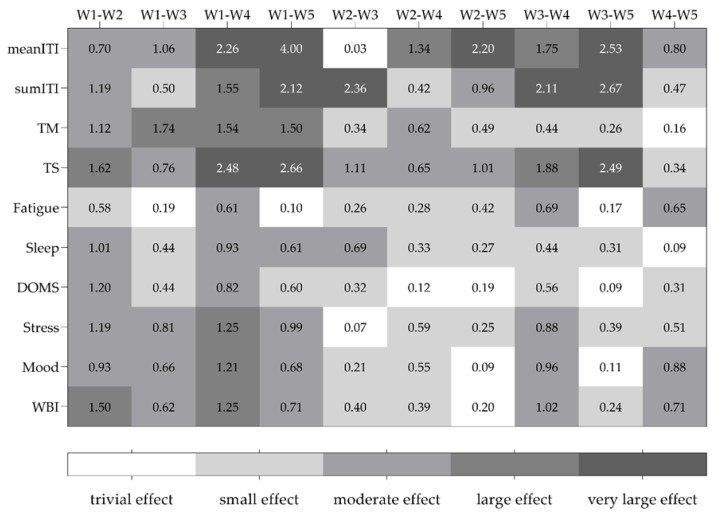
Multiple comparisons between weeks in internal training intensity and well-being status measures expressed as the magnitude of effect size; meanITI—average weekly internal intensity; sumITI—accumulated weekly internal intensity; TM—training monotony; TS—training strain; DOMS—delayed onset of muscle soreness; WBI—well-being status index; W—week.

Measures of magnitude for multiple comparisons between weeks in ITI and well-being status measures are presented in Figure 5. Large to very large effects are found for both meanITI and sumITI for the week 1 vs. week 5 and week 3 vs. week 5 pairwise comparisons. Lower values (trivial to moderate effect) were found for the well-being status domains sleep and DOMS.

**Figure 6:**
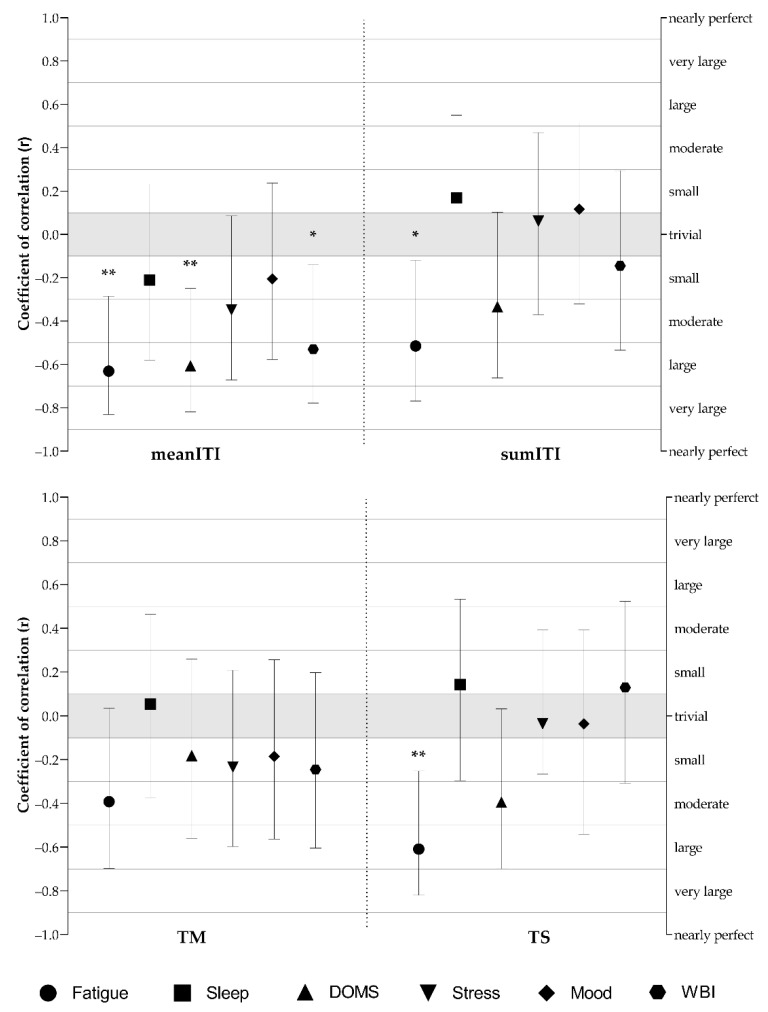
Coefficients of correlation (r) determined between internal intensity and well-being status measures; meanITI—average weekly internal intensity; sumITI—accumulated weekly internal intensity; TM—training monotony, TS—training strain; DOMS—delayed onset of muscle soreness; WBI—well-being status index. Error bars represent 90% confidence limits (CL); *—significance at *p* < 0.05; **—significance at *p* < 0.01.

Pearson’s correlation coefficients between ITI and well-being status measures are presented in Figure 6. Large negative correlations between mean ITI and fatigue (r = −0.63, *p* = 0.002), DOMS (r = −0.61, *p* = 0.003), and WB (r = −0.53, *p* = 0.011), sumITI, and fatigue (r = −0.51, *p* = 0.014), and TS and fatigue (r = −0.61, *p* = 0.003) were found. Moderate to small correlations were found between TM and TS with well-being measures, but without statistical significance. Moreover, small but non-significant correlations were found between CMJ performance and well-being status measures.

## 4. Discussion

This study aimed to assess the ITI, well-being status, and jumping performance in professional soccer players during the five weeks of the pre-season phase preceding the national league season. Additionally, the study aimed to determine the relationship between ITI indices, CMJ performance, and well-being status measures. The main findings of this study were as follows: (i) most TE-TA and S&C training sessions were performed at low intensity, while the proportion of matches played at high intensity was over 90%; (ii) lower daily WBI values were observed after days of higher ITI; (ii) CMJ performance progressively increased over five weeks; (iii) in weeks with lower values of ITI, higher CMJ performance was observed; (iv) significant differences were found for all ITI and well-being status measures across the weeks, with TM and TS being lowest in the final week of the pre-season phase; (v) large negative association was observed between average ITI and the well-being status domains of fatigue, DOMS, and WB, as well as between accumulated ITI and TS and fatigue.

Players spent the majority of their TE-TA and S&C sessions in the low intensity zone, with a proportion of approximately 53% and 80%, respectively. Sessions at moderate intensity took accounted for approximately 28% of the time for TE-TA trainings and 20% of the time for S&C trainings, while high intensity was determined only for the TE-TA trainings (approximately 20%) (Figure 1). Players’ performances in the combined TE-TA and S&C training sessions occurred at approximately 61, 25, and 14% in the low, moderate, and high intensity ranges, respectively. Training intensity distribution in this study across the five weeks was almost identical to those reported by [32], in which U20 soccer players completed over 60% of their training sessions at low intensity, while moderate and high intensity constituted 25% and 15% of their total trainings, respectively. A more balanced distribution was reported for Norwegian soccer [33] and Australian rugby [34] players; hoverer, these investigations included the in-season (competition) phase, in which official games took place. Furthermore, the high proportion of Z1 could be explained by the fact that all training sessions were computed for analysis in this real-world scenario (including recovery sessions with low training intensity).

During the five weeks of the pre-season phase, the accumulated ITI (average accumulated weeklyITI: 2439.9 ± 224.7 a.u.) were similar to those reported for professional senior soccer players [35,36], but higher than those reported for junior soccer players [23,37]. Significant week-to-week changes in average weekly ITI (0.5 to 26%) were determined between weeks (Table 2), indicating that there was progression in the stimulus, as well as inter-week variability. Lack of progression is associated with injury risk and poor athlete performance [38]. Moreover, stabilization of the intensity may be one factor contributing to a performance plateau. Athletes should not adopt the variability and progression of the intensity based on the general absence of changes in the training intensity throughout the week [39]. Similarly, significant week-to-week changes found in well-being status measures (Table 2) clearly show that well-being and physical adaptations to training appear to be significantly influenced by absolute training intensities collected over time and their distribution over the weeks [40]. It is well known that subjective well-being commonly deteriorates with an acute increase in training intensity and improves with an acute reduction in training intensity [41]. The TM and TS parameters (derived from sRPE) are important factors related to negative adaptations to training [42]. In this study, the TM and TS showed a significant decrease over the weeks (Table 2), with the highest values of 2.2 and 6656.3 in the first week, respectively. A similar TM pattern was observed in senior [15] and junior soccer players [32,36] during the same period. A decrease in TM during tapering is associated with improvement in physical match activity and therefore, represents a good indicator of the state and readiness of the players [43]. However, recent systematic review reflected that acute:chronic workload ratio (ACWR), along with TM, should be used to better explain the physical capacities of players [44]. Given that the study period was five weeks, the ACWR was not considered. Within-week variations for the TM (CV% from 13.2 to 16.8) and TS (CV% from 16.4 to 32.5) indicate individualized ITI responses, although the training program was similar for all players; however, this can be related to the different physical demands of each playing position [32]. 

CMJ performance over the period of investigation showed a progressive increasing trend (Figure 4). CMJ height in weeks 4 and 5 was most likely to very likely higher when compared to week 1 (baseline). In week 2, athletes showed a slight decrease in CMJ performance, but these changes were not meaningful. The progressive increase was probably induced by the reduction of the training intensity in the following weeks of the training program. In a study by Cruz et al. [40], CMJ performance progressively increased when the training intensities were reduced over the nine weeks of the pre-season phase in basketball. Moreover, similar observations were also reported for soccer [45], rugby [10], volleyball [46] and futsal [47].

The secondary aim of this study was to examine the dose–response relationship between ITI and the variations in well-being status measures (Figure 6). Results revealed that fatigue largely correlated with average and accumulated weekly ITI and TS. Moreover, DOMS and overall WB score were largely correlated with average ITI. Interestingly, non-significant negative small to moderate correlations were observed between TM and all well-being status measures. Similar results were found for soccer [48] and basketball players [49], where fatigue and DOMS had higher correlations with ITI compared with other well-being categories. No relationships with ITI are specifically confirmed for sleep, where the authors found significant correlations with fatigue, DOMS, and overall well-being score, but not with athletes’ sleep quality [7,18]. Additionally, variations in sleep quality, fatigue, and DOMS were more sensitive to acute rather than chronic training intensity or TM [50]. DOMS and perceived fatigue may be related to each other and are associated with the physical strain players experience during training sessions, therefore presenting high sensitivity to variations in ITI [49]. However, deeper analysis is required, as perceived well-being indices can be influenced by other environmental factors, such as high altitude, religious practices, league ranking, and match importance [51], which are not controlled in this study. This is especially true for sleep, stress, and mood, which represent the psycho-cognitive component of the well-being status and directly influence each other. Reducing stress may enhance sleep quality, whereas sleep issues may have a negative impact on the stress system and attenuate endocrine stress responses [52]. Moreover, the main contextual factors to consider when identifying stress sources include negative organizational systems and cultures, concerns about performance expectations and standards, career development issues, negative aspects of interpersonal relationships, the demanding nature of work itself, and issues regarding the work/non-work interface [53].

Since the CMJ test is reliable and well-accepted by professional athletes, it is useful for identifying and quantifying fatigue in field conditions [54]. Despite the fact that the CMJ height and well-being scores varied with training intensities, there was only a weak, non-significant association between the two. This is in contrast with the previous studies in which a significant relationship was determined between the two [55,56]. Similar findings were reported in a study regarding wrestlers, in which the authors suggest that coaches should analyze WBI instead of single well-being variables [57]. Another study conducted on baseball players reports that a five-item well-being questionnaire demonstrated consistency and efficiency; however, it did not reflect positive or negative changes in CMJ performance [58]. Subjective and objective measures assess different aspects of fatigue. This demonstrates that several subjective and objective factors need to be monitored for making informed judgments, instead of concentrating on either subjective or objective measurements alone, which has crucial implications for monitoring fitness and fatigue.

### 4.1. Study Limitations

Although conducted in a real-world scenario, this study has some limitations. The observations were conducted during a professional pre-season phase; therefore, it was impossible to form a control group or evaluate as many parameters as would have been desirable. There were no objective internal or external intensity measurements applied and sRPE was the sole internal training intensity indicator, which may have inhibited a more accurate measurement of the training’s physiological effects. As perceived effort scales have been found to be highly linked with heart rate measurements and GPS information, it would be interesting to include these variables to assess players’ responses to the training stimulus. Moreover, the integrations of s-RPE with GPS data provide a better approach to obtaining knowledge about TM [59]. Additionally, it is known that poor sleep and nutritional habits can have negative effects on players’ performance [60,61]; however, due to the nature of the study, these parameters could not be monitored.

### 4.2. Practical Applications

While some limitations were disclosed, this study possesses good ecological validity, as it was conducted with professional soccer players during the pre-season phase before the competitive season. Complementary research should be conducted to close the gap between the theory and practice of training periodization. Coaches and others involved in developing training programs should be aware of the significance of perceived exertion in effectively monitoring soccer players’ well-being. Assessing relative training intensities after a training session could be critical in determining whether neuromuscular fatigue and DOMS, rather than disturbances in sleep, stress, and mood, will occur in players within 24 h. Although it is challenging to apply an individual approach, it is recommended that different modalities should be used because each player responds differently to training stimuli. Monitoring these measures during the pre-season assists coaches in preparing players for the demands of matches during the in-season phase.

## 5. Conclusions

Lower daily WB values were observed after days of higher ITI; likewise, decrements in weekly ITI induced positive CMJ performances, with a progressive trend over the weeks. Significant week-to-week changes in ITI measures indicated that there was progression in the stimulus and inter-week variability, which possibly reduced injury risk. Moreover, inter-week variability in WB components clearly showed that well-being and physical adaptations to training appeared to be significantly influenced by absolute training intensities. Only the meaningful correlations found between ITI and fatigue and DOMS support previous findings of reduced sensitivity for psycho-cognitive perceptions of sleep, stress, and mood. Neuromuscular fatigue and its relationship with well-being should be used as a complementary analysis, with an increased number of objective and subjective measures included in training monitoring. Overall, this study highlights the utility and simplicity of monitoring tools to improve athletes’ performance. Coaches and sports scientists can more effectively create and modify their training programs by using descriptions of the typical ITI experienced by professional soccer players during preparation for the competitive season. Lastly, a balanced training response/recovery ratio is important to effectively increase players’ physical performance.

## Figures and Tables

**Table 1:** Description of the training program over the 5 weeks of the pre-season phase.

Measure		Week 1	Week 2	Week 3	Week 4	Week 5
TE-TA sessions (*n*)		5	4	5	4	5
time	Σ	430	320	320	275	330
	mean	86	80	80	68.8	66
	SD	14.7	22.7	17.3	6.3	15.6
S&C sessions (*n*)		3	3	2	1	/
time	Σ	175	200	120	60	/
	mean	58.3	66.7	60	60	/
	SD	2.9	2.9	/	/	/
Matches (*n*)		1	1	2	2	2

**Table 2:** Internal training intensity and well-being status measures of soccer players over the 5 weeks of the pre-season phase.

Measure		W1	W2	W3	W4	W5
meanITI	mean	549.3	483.2 ^1^	485.6 ^1^	356.1 ^1,2,3^	294.3 ^1,2,3,4^
	SD	52.4	84.1	50.3	57.2	44.7
sumITI	mean	3035.7	2259.1 ^1^	3310.9 ^2^	1946.4 ^1,2,3^	1647.3 ^1,3^
	SD	409.9	536.0	416.6	537.9	367.9
TM	mean	2.2	1.8 ^1^	1.7 ^1,2^	1.5 ^1,3^	1.6 ^1,3^
	SD	0.3	0.3	0.2	0.3	0.2
TS	mean	6656.3	4104.4 ^1^	5565.9 ^1,2^	3022.8 ^1,3^	2599.9 ^1,2,3^
	SD	1093.3	1322.3	1046.4	982.7	622.2
fatigue	mean	3.3	3.1	3.2	2.9 ^3^	3.3
	SD	0.4	0.4	0.5	0.4	0.4
sleep	mean	4.1	3.7 ^1^	3.9 ^2^	3.7 ^1^	3.8
	SD	0.3	0.4	0.3	0.4	0.4
DOMS	mean	3.6	3.2 ^1^	3.4	3.2 ^1^	3.3
	SD	0.4	0.4	0.4	0.4	0.3
stress	mean	4.2	3.8 ^1^	3.8 ^1^	3.5 ^1,4^	3.7 ^1^
	SD	0.4	0.4	0.4	0.6	0.4
mood	mean	4.3	4.0 ^1^	4.0	3.7 ^1,4^	4.0 ^1^
	SD	0.4	0.4	0.4	0.4	0.3
WBI	mean	19.3	17.7 ^1^	18.3	17.1 ^1,3^	18.1 ^1,5^
	SD	1.4	1.7	1.4	1.7	1.2

Legend: meanITI—average weekly internal intensity; sumITI—accumulated weekly internal intensity; TM—training monotony; TS—training strain; DOMS—delayed onset of muscle soreness; WBI—well-being status index; W—week; statistically different from ^1^ W1, ^2^ W2, ^3^ W3, ^4^ W4, ^5^ W5.

## Data Availability

Data can be provided on reasonable request.
